# Salvianolic acid B prevents epithelial-to-mesenchymal transition through the TGF-β1 signal transduction pathway *in vivo *and *in vitro*

**DOI:** 10.1186/1471-2121-11-31

**Published:** 2010-05-05

**Authors:** Qing-Lan Wang, Yan-Yan Tao, Ji-Li Yuan, Li Shen, Cheng-Hai Liu

**Affiliations:** 1 Institute of Liver Diseases, Shuguang Hospital Affiliated to Shanghai University of Traditional Chinese Medicine, 528 Zhangheng Road, Shanghai, China; 2 Key Laboratory of Liver and Kidney Diseases (Shanghai University of Traditional Chinese Medicine), Ministry of Education, 528 Zhangheng Road, Shanghai, China; 3 E-Institute of TCM Internal Medicine, Shanghai Municipal Education Commission, 1200 Cailun Road, Shanghai, China

## Abstract

**Background:**

Salvianolic Acid B (Sal B) is a water-soluble component from Danshen (a traditional Chinese herb widely used for chronic renal diseases) with anti-oxidative and cell protective properties. Sal B also has potential protective effects on renal diseases. Tubular epithelial cells can undergo epithelial-to-mesenchymal transition (EMT), which plays an important role in the pathogenesis of renal interstitial fibrosis (RIF) and is mainly regulated by TGF-β1/Smads pathway. The aims of the study are to investigate the effect of Sal B on tubular EMT *in vivo *and *in vitro*, and to elucidate its underlying mechanism against EMT related to TGF-β1/Smads pathway.

**Results:**

For *in vivo *experiments, RIF was induced in rats by oral administration of HgCl_2 _and prophylaxised with Sal B and vitamin E. The protein expression of E-cadherin was down-regulated, while the expression of α-SMA, TGF-β1, TβR-I, p-Smad2/3 and the activity of matrix metalloproteinase-2 (MMP-2) were up-regulated in kidneys of model rats when compared with those of normal rats. In contrast, Sal B and vitamin E significantly attenuated the expression of α-SMA, TGF-β1, TβR-I, p-Smad2/3, and MMP-2 activity, but increased E-cadherin expression. For *in vitro *experiments, HK-2 cells were incubated with TGF-β1 to induce EMT, and the cells were co-cultured with 1 and 10 μM Sal B or SB-431542 (a specific inhibitor of TβR-I kinase). TGF-β1 induced a typical EMT in HK-2 cells, while it was blocked by Sal B and SB-431542, as evidenced by blocking morphologic transformation, restoring E-cadherin and CK-18 expression, inhibiting α-SMA expression and F-actin reorganization, and down-regulating MMP-2/9 activities in TGF-β1 mediated HK-2 cells. Furthermore, Sal B and SB-431542 profoundly down-regulated the expressions of TβR-I and p-Smad2/3 but prevented the decreased expression of Smad7 in TGF-β1 stimulated HK-2 cells.

**Conclusions:**

Sal B can prevent tubular EMT in the fibrotic kidney induced by HgCl_2 _as well as HK-2 cells triggered by TGF-β1, the mechanism of Sal B is closely related to the regulation of TGF-β1/Smads pathway, manifested as the inhibition of TGF-β1 expression, suppression of TβR-I expression and function, down-regulation of Smad2/3 phosphorylation, and restoration of the down-regulation of Smad7, as well as inhibition of MMP-2 activity.

## Background

Renal interstitial fibrosis (RIF) is a common manifestation in progressive renal diseases leading to functional deterioration and eventual loss of renal function, irrespective of the nature of the initial renal injury [1]. Activation of tubulointerstitial myofibroblast leads to the production of excessive extracellular matrix with a predominance of interstitial collagens, and plays a critical role in the progression of chronic renal diseases [2]. Emerging evidence suggests that myofibroblasts can be derived from tubular epithelial cells through the process of epithelial-to mesenchymal transition (EMT) during the progression to renal fibrosis [3]. Transforming growth factor-beta1 (TGF-β1) has been proposed to be the major regulator in inducing EMT and renal fibrosis [4], mainly via the TGF-β/Smads signal transduction pathway [5]. Despite EMT contributes to the disease progression, several studies have suggested that EMT of the tubular epithelial cell can be reversible [6].

**Figure 1:**
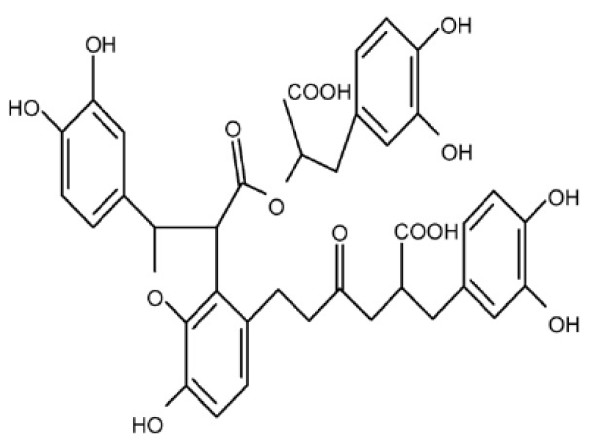
**The chemical structure of Sal B**.

Danshen (Radix Salviae Miltiorrhizae, SM) is a popular traditional Chinese herb widely used in treating cardiovascular, renal, and hepatic diseases by improving circulation and resolving stasis [7,8]. Salvianolic acid B (Sal B) (Figure 1), also known as lithospermates B or tanshinoate B, is a major water soluble component extracted from SM, which was well recognized as an antioxidative agent and free radical scavenger, involved in the protection of various cells, including nerve cells and hepatocytes [9-12]. It was reported that SM and its aqueous extract, Sal B, produced a good effect in treating patients and animals with chronic renal diseases. They could improve renal function in uremic rats through the activation of kallirein and the promotion of prostaglandin E2 production [13-15]. Moreover, recent research showed that Sal B can prevent TGF-β1-induced EMT of human kidney cell lines *in vitro *[16]. Our previous studies showed that Sal B had obvious therapeutic effects against liver fibrosis in patients with chronic hepatitis B [17] and animal models induced by dimethylnitrosamine [18], and its mechanism was closely associated with the inhibition of hepatic stellate cell activation through the regulation of TGF-β/Smads signaling [19]. Moreover, our recent study found that Sal B can prevent RIF induced by mercuric chloride (HgCl_2_) in rats, with the mechanism associated with anti-oxidative injury [20].

Although the pivotal role of tubular EMT in RIF is widely accepted [21], a recent controversial report discussing the epithelial origin for myofibroblast *in vivo *[22] suggests that EMT *in vivo *may be different from EMT *in vitro*. Therefore it is definitely worthwhile to evaluate the effect of Sal B on tubular EMT *in vivo *as well as *in vitro *and probe its underlying mechanism of action in order to obtain more solid evidence of Sal B's action on tubular EMT and better understand its mechanism. In this study, we observed the effects of Sal B on renal tubular EMT *in vivo *and *in vitro *and investigated Sal B's underlying mechanism of action against EMT in renal tubular epithelial cells relating to the TGF-β/Smads signal pathway. The results demonstrate that Sal B can prevent tubular EMT in the fibrotic kidney induced by HgCl_2 _as well as human proximal tubular epithelial cells (HK-2 cell line) after being triggered by TGF-β1. The mechanism of action is closely related to the regulation of the TGF-β1 signaling pathway via the inhibition of TGF-β1 expression, suppression of TGF-β type I receptor (TβR-I) expression and function, down-regulation of Smad2/3 phosphorylation, and restoration of the down-regulation of Smad7 by TGF-β1, as well as the inhibition of matrix metalloproteinase 2 (MMP-2) activity in diseased kidneys.

## Results

### 1. Sal B ameliorated renal interstitial fibrosis induced by HgCl_2 _in rats

**Figure 2:**
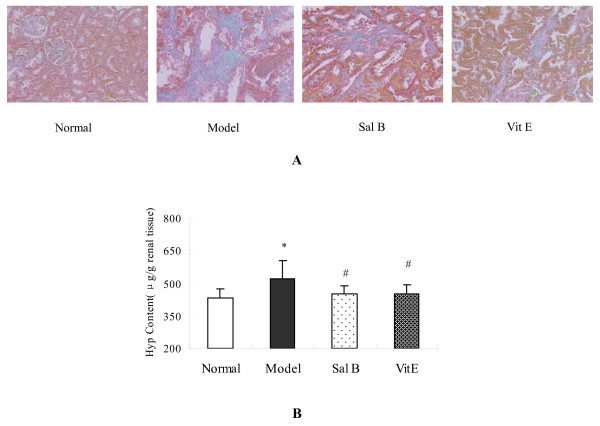
**Sal B ameliorated renal interstitial fibrosis induced by HgCl_2 _in model rat**. (A) Masson's trichrome staining for assessing renal interstitial fibrosis. HgCl_2 _induced an increase of collagen in kidney section. Sal B and Vit E treatments reduced collagen deposition. (B) Kidney Hyp content determined by Jamall's method. Hyp content of kidneys was increased significantly in the model group as compared with the normal group. In contrast, Sal B and Vit E treatments significantly decreased kidney Hyp content.

The excessive and disorganized deposition of collagens is a major pathogenic feature of fibrotic diseases. HgCl_2 _induced an increased amount of collagen formation in the Masson's trichrome-stained kidney sections. In contrast, collagen levels diminished in Sal B and Vitamin E (Vit E) treated fibrotic kidneys (Figure 2A.).

Hydroxyproline (Hyp) content is a specific marker for collagen synthesis. The results showed that Hyp content in kidney increased significantly in the model group when compared with the normal group. In contrast, Sal B and Vit E treatments significantly decreased kidney Hyp content of model rats. There was no significant difference in Hyp content between Sal B and Vit E groups (Figure 2B.).

### 2. Effects of Sal B on the expressions of alpha smooth muscle actin (α-SMA) and E-cadherin in kidneys of model rats

**Figure 3:**
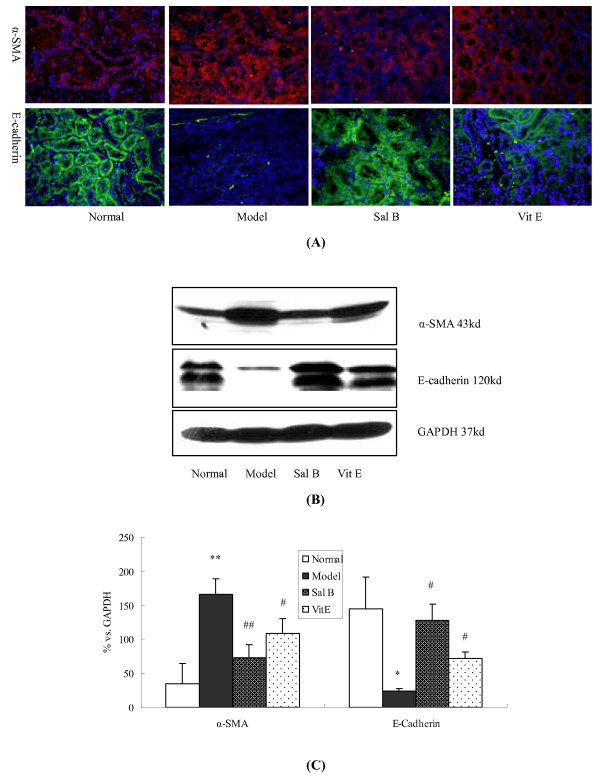
**The Effects of Sal B on α-SMA and E-Cadherin expression in kidneys of model rats**. (A) Immunofluorescence staining of α-SMA and E-cadherin in kidneys. Increased labeling of α-SMA in the tubule was observed in model rats, while it was noted to be decreased by Sal B treatment. The E-cadherin labeling intensity was decreased in model rats, whereas the intensity was maintained in the Sal B treatment group (B) Western blot analyses for α-SMA and E-cadherin expression in renal tissues. Significantly increased α-SMA expression and decreased E-cadherin expression were observed in rats of the model group. Sal B treatment attenuated HgCl_2_-induced upregulation of α-SMA expression. Moreover, it significantly inhibited the HgCl2-induced decrease of E-cadherin expression. (C) Graphic presentation of the relative expression of α-SMA and E-cadherin. The values are represented as the density of α-SMA or E-cadherin *vs *GAPDH (%). **P < 0.01 *vs *normal; *P < 0.05 *vs *normal; #*P *< 0.05 *vs *Model; # #*P *< 0.01 *vs *Model.

Immunohistochemistry analysis revealed that the expression of α-SMA was very weak in the renal tubule and interstium of normal kidneys, whereas it was prominent in model rats. On the other hand, epithelial cell marker, E-cadherin, was markedly decreased in the tubular epithelium of model groups compared with the normal group. Sal B treatment attenuated the increased expression of α-SMA in fibrotic kidneys. Moreover, E-cadherin expression was maintained in response to Sal B treatment (Figure 3A). Therefore, the disruption of the tubular basement membrane was ameliorated with Sal B treatment. Vit E had the same effects. This observation may indicate that Sal B inhibits the process of EMT in fibrotic kidneys, as characterized by the transition from the epithelial to the mesenchymal phenotype.

Consistent with the immunohistochemistry studies, Western blot analysis revealed increased α-SMA and decreased E-cadherin expression in the kidneys of model group rats when compared with the normal group. In contrast, Sal B and Vit E treatments significantly inhibited the up-regulation of α-SMA and the down-regulation of E-cadherin. Howerer, the effect of Sal B was significantly more pronounced than Vit E. (Figure 3B, C).

### 3. Sal B treatment attenuated the increase in TGF-β1, TβR-I, and Smad2/3 phosphorylation in kidneys of model rats

**Figure 4:**
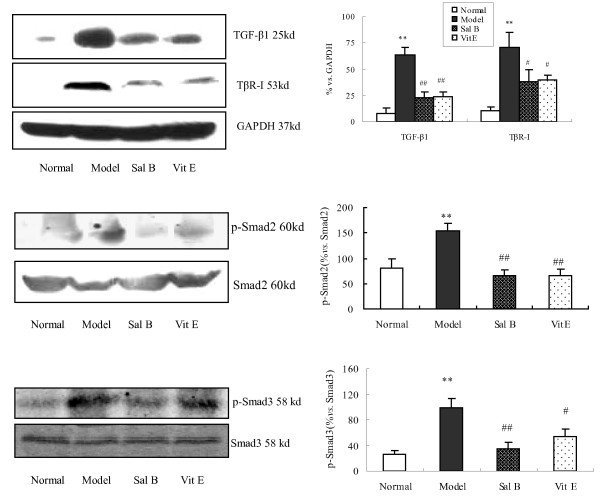
**The Effect of Sal B on TGF-β1, TβRI, Smad2, p-Smad2 and p-Smad3 expression in kidneys of model rats**. Western blot analysis showed significantly increased TGF-β1, TβR-I, p-Smad2 and p-Smad3 expressions in rats of the model group. In contrast, Sal B treatment attenuated HgCl_2_-induced upregulation of TGF-β1, TβR-I, p-Smad2 and p-Smad3 expressions. The values are represented as the density of TGF-β1 or TβR-I *vs*. GAPDH (%), or p-Smad2 *vs*. Smad2 (%), or p-Smad3 *vs*. Smad3 (%). **P < 0.01 *vs*. normal; *P < 0.05 *vs*. normal; ## *P *< 0.01 *vs*. model; #*P *< 0.05 *vs*. model.

TGF-β1/Smads signaling is known to play a major role in the process of TGF-β1-induced EMT. The results demonstrated that there was no significant change in Smad2 and Smad3 expression between each group, while the expression of phosphorylated Smad2 and Smad3, TGF-β1, and TβR-I was significantly increased in kidneys of rats in the model group when compared with those of the normal group. Conversely, Sal B treatments significantly attenuated the up-regulation of TGF-β1, TβR-I, p-Smad2, and p-Smad3 (Figure 4).

### 4. Sal B down-regulated the activity of MMP-2 in kidneys of model rats

**Figure 5:**
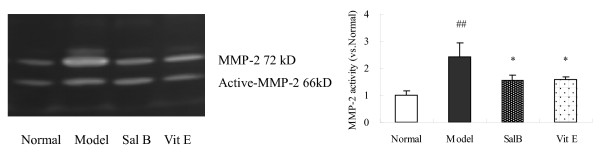
**The effect of Sal B on the activity of MMP-2 in kidneys of model rats**. The activity of MMP-2 was assessed by gelatin zymography. The results showed the activity of MMP-2 was increased in rats of model group when compared with that of normal group. Sal B significantly down-regulated the activity of MMP-2. **P < 0.01 *vs*. normal; # *P *< 0.05 *vs*. Model.

Disruption of the tubular basement membrane (TBM) is a key event essential for EMT at the cellular level, and MMP-2 can degrade TBM proteins such as type IV collagen, leading to the initiation of tubular EMT. The activity of MMP-2 in kidney tissue was assessed using gelatin zymography. As shown in Figure 5, the activity of MMP-2 was increased in rats of the model group when compared with that of the normal group. Sal B and Vit E significantly down-regulated the activity of MMP-2. (Figure 5.)

### 5. Effects of Sal B on cell viability and toxicology of HK-2 cells

**Figure 6:**
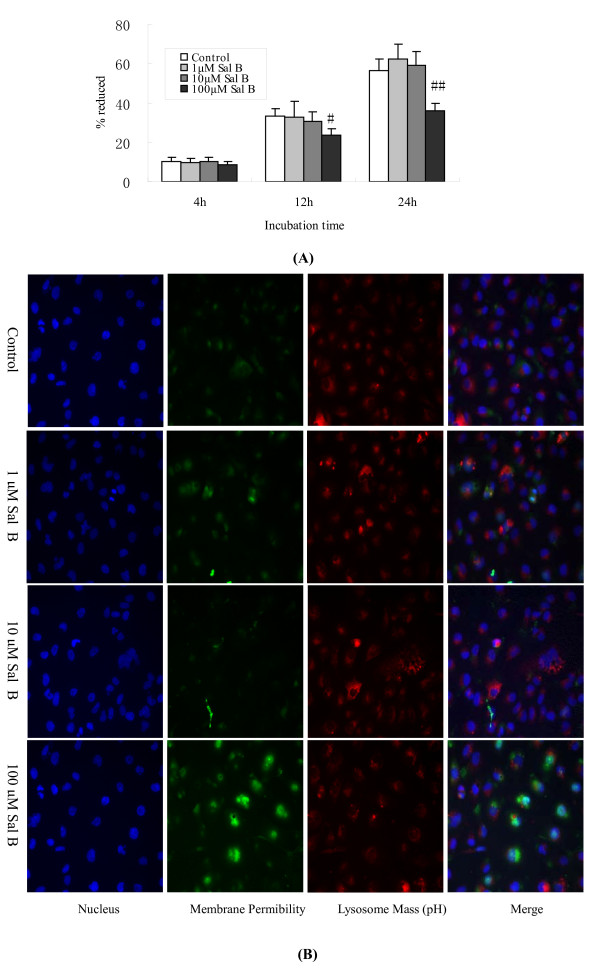
**Effects of Sal B on cell viability and toxicology in HK-2 cells**. (A) Cell viability was determined by the alamarBlue assay. The results were expressed as a percentage in the reduction of alamarBlue. 1 μM and 10 μM of Sal B had no significant effect (*P *> 0.05) on cell viability during each incubation time period, whereas 100 μM of Sal B decreased the viability of HK-2 cells by 11% (*P *< 0.05) and 20% (*P *< 0.05), when compared with control cells after 12 h and 24 h of incubation, respectively. (B) Cell toxicology was determined by high content screening (HCS) assay. HK-2 cells were plated in a 96-well plate at a density of 4,000 cells/well. Cells were incubated with 1-100 μM of Sal B for 24 h and then stained with 1 × MPCT1 Fluor Dye. Cell images from the various fluorescent dye stains were taken by HCS (×200). There were no obvious changes in nuclear morphology, membrane permeability, or lysosomal mass/pH after incubation with 1 μM and 10 μM of Sal B when compared with control cells. However, 100 μM of Sal B presented as an obvious cytotoxicity to cells. As shown by the indicator dye, there are fewer nuclei and an increase in membrane permeability.

Cell viability was determined by the alamarBlue assay. The results were expressed as the percentage of reduction in alamarBlue. 1 μM and 10 μM of Sal B had no significant effect (*P *> 0.05) on cell viability during each incubation period, whereas 100 μM of Sal B decreased the viability of HK-2 cells by 11% (*P *< 0.05) and 20% (*P *< 0.05) when compared to control cells after 12 h and 24 h of incubation, respectively (Figure 6A).

To investigate the cytotoxicity of Sal B on HK-2 cells, cells were treated with a wide range of Sal B doses from 1 μM to 100 μM. After 24 h of incubation, we found no obvious changes in nuclear morphology, membrane permeability, or lysosomal mass/pH with 1 μM and 10 μM of Sal B treatments in comparison with control cells. However, 100 μM of Sal B showed an obvious cytotoxicity to cells, reflected by fewer nuclei and an increase in the membrane permeability as shown by the indicator dye. Therefore, 1 μM and 10 μM of Sal B were considered to be safe doses for treating cells (Figure 6B).

### 6. Sal B treatment blocked TGF-β1-induced EMT in HK-2 cells

#### (1) Sal B prevented the loss of E-Cadherin and Cytokeratin-18 (CK-18) expressions and inhibited α-SMA expression induced by TGF-β1 in HK-2 cells

**Figure 7:**
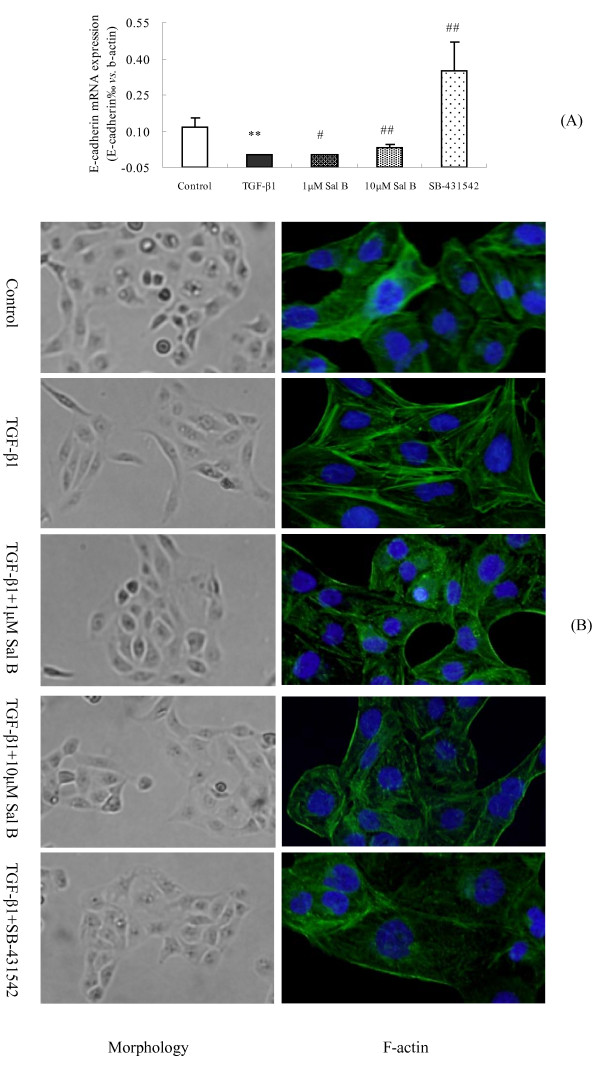
**The effects of Sal B on E-cadherin mRNA expression, cell morphologic transformation and F-actin reorganization induced by TGF-β1 in HK-2 cells**. (A) The effects of Sal B on E-cadherin mRNA expression. Real-time PCR analysis showed that TGF-β1 significantly suppressed E-cadherin mRNA expression in HK-2 cells, while 1 μM and 10 μM of Sal B prevented the loss of its expression (B) The effects of Sal B on cell morphology transformation and F-actin reorganization. HK-2 cells were incubated with serum-free medium or treated with (1) 2.5 ng/ml TGF-β1, (2) 2.5 ng/ml TGF-β1 plus 1 μM of Sal B, (3) 2.5 ng/ml TGF-β1 plus 10 μM of Sal B, (4) 2.5 ng/ml TGF-β1 plus 10 μM of SB-431542 for 24 h. HK-2 cells changed from a cuboidal to a spindle shape in response to TGF-β1, whereas treatment with 1 μM and 10 μM Sal B and SB-431542 blocked this morphologic transformation (×200). Representative micrographs of FITC-conjugated phalloidin staining showed significanat F-actin reorganization and the formation of abundant long stress fibers in HK-2 cells induced by TGF-β1 were formed. 1 μM and 10 μM of Sal B and SB-431542 significantly blocked these processes.

E-cadherin is a tubular epithelial cell-cell adhesion receptor that is essential for the formation and maintenance of renal epithelial homeostasis and architecture [23,24]. We found that TGF-β1 significantly suppressed E-cadherin mRNA expression in HK-2 cells, while 1 and 10 μM of Sal B prevented the loss of its expression (Figure 7A).

**Figure 8:**
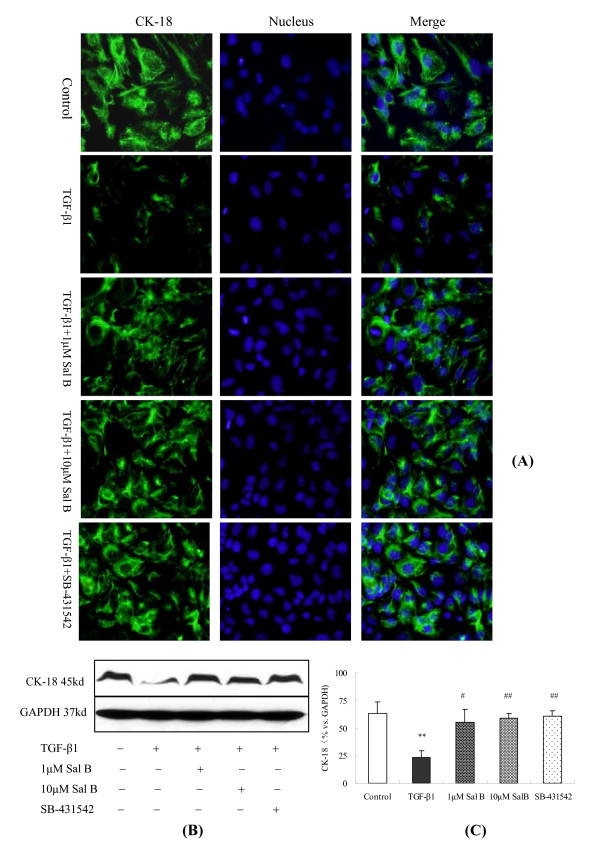
**Sal B depressed CK-18 expression induced by TGF-β1 in HK-2 cells**. HK-2 cells were cultured in complete medium containing 5% FBS for 18 h. Thereafter, the cells were kept in serum-free medium or treated with (1) 2.5 ng/ml TGF-β1, (2) 2.5 ng/ml TGF-β1 plus 1 μM of Sal B, (3) 2.5 ng/ml TGF-β1 plus 10 μM of Sal B, (4) 2.5 ng/ml TGF-β1 plus 10 μM of SB-431542 for 24 h. (a) Immunofluorescence staining (×200) showed decreased immunolabeling intensity of CK-18 after TGF-β1 treatment. This effect was depressed by 1 μM and 10 μM of Sal B as well as SB-431542 treatments. The blue-colored stain is nuclear counterstaining with Hoechst 33258. (B) Western blot analysis for CK-18. CK-18 expression was decreased when cells were exposed to TGF-β1, whereas 1 μM and 10 μM of Sal B and SB-431542 significantly attenuated the TGF-β1-induced up-regulation of α-SMA. (C) Graphic presentation of the relative expression of CK-18. The values are represented as 100% *vs*. control. **P < 0.01 *vs*. control; #*P *< 0.05 *vs*. TGF-β1, # #P < 0.01 *vs*. TGF-β1.

Consistent with the alteration in E-cadherin mRNA expression, both immunofluorescence and Western blot analyses showed that CK-18, an epithelial cell marker, was significantly down-regulated after TGF-β1 treatment and was then reversed by 1 μM and 10 μM of Sal B treatments (Figure 8).

**Figure 9:**
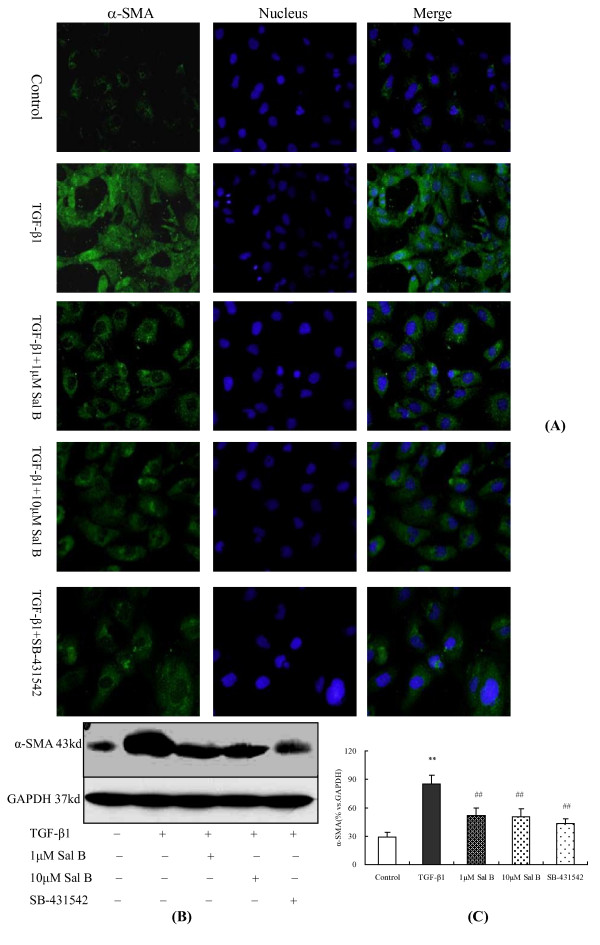
**Sal B blocked α-SMA expression induced by TGF-β1 in HK-2 cells**. HK-2 cells were cultured in complete medium containing 5% FBS for 18 h. Thereafter, the cells were kept in serum-free medium or treated with (1) 2.5 ng/ml TGF-β1, (2) 2.5 ng/ml TGF-β1 plus 1 μM of Sal B, (3) 2.5 ng/ml TGF-β1 plus 10 μM of Sal B, (4) 2.5 ng/ml TGF-β1 plus 10 μM of SB-431542 for 24 h. (A) Immunofluorescence staining (×200) shows the increased labeling intensity of α-SMA observed after TGF-β1 treatment, while its increase becomes reversed after 1 μM and 10 μM Sal B and SB-431542 treatments. The blue-colored stain is nuclear counterstaining with Hoechst 33258. (B) Western blot analysis for α-SMA. α-SMA expression was increased when the cells were exposed to TGF-β1, whereas treatment with 1 μM and 10 μM Sal B and SB-431542 significantly attenuated the up-regulation of α-SMA by TGF-β1. (C) Graphic presentation of the relative expression of α-SMA. The values are represented as 100% *vs*. GAPDH. **P <0.01 *vs*. control; # #*P *< 0.01 *vs*. TGF-β1.

On the other hand, α-SMA (a specific myofibroblast marker) expression was increased significantly after TGF-β1 incubation, as detected by immunofluorescence and Western blot analyses, while 1 μM and 10 μM of Sal B treatments significantly attenuated its expression (Figure 9).

#### (2) Sal B blocked the transformation of cell morphology and F-actin reorganization induced by TGF-β1

Concomitant with the alterations in E-cadherin, CK-18, and α-SMA expressions as described above, we found that 1 μM and 10 μM of Sal B blocked tubular epithelial cells from taking on a myofibroblastic appearance induced by TGF-β1. HK-2 cells possess a cobblestone appearance when grown in culture, but they become elongated and take on a spindle shape in response to TGF-β1. Co-incubation of Sal B with TGF-β1 restored the epithelial morphology of these cells (Figure 7B).

The actin cytoskeleton plays an important role in defining cell shape and morphology [25]. In this study, we also examined F-actin reorganization during the conversion from tubular epithelium to myofibroblasts after TGF-β1 administration. The results revealed that TGF-β1 induced significant F-actin reorganization, forming abundant long stress fibers in HK-2 cells. 1 μM and 10 μM of Sal B blocked these processes (Figure 7B).

#### (3) Sal B down-regulated MMP-2 and MMP-9 activation induced by TGF-β1 in HK-2 cells

**Figure 10:**
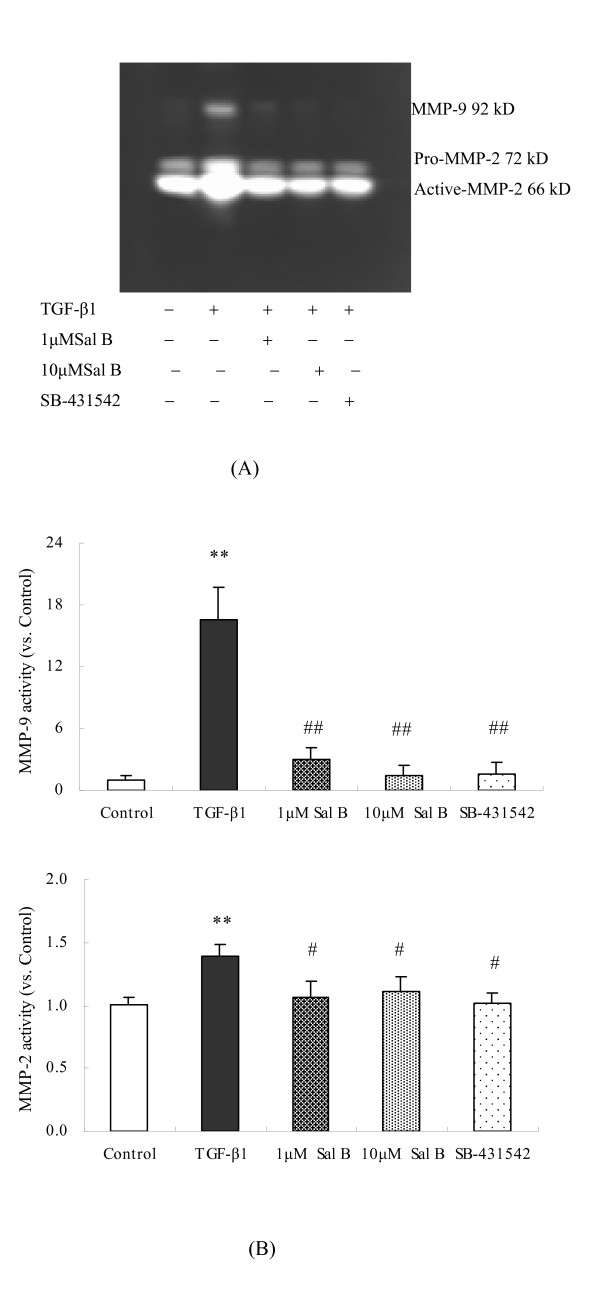
**Sal B down-regulated MMP-2 and MMP-9 activation induced by TGF-β1 in HK-2 cells**. HK-2 cells were incubated with serum-free medium or treated with (1) 2.5 ng/ml TGF-β1, (2) 2.5 ng/ml TGF-β1 plus 1 μM Sal B, (3) 2.5 ng/ml TGF-β1 plus 10 μM Sal B, (4) 2.5 ng/ml TGF-β1 plus 10 μM SB-431542 for 24 h. The activation of MMP-2 and MMP-9 derived from cells in the conditioned media were analyzed by gelatin zymographic analysis. The results show that TGF-β1 induced pro-MMP-2, the activation of pro-MMP-2, and MMP-9 secretion. 1 μM Sal B, 10 μM Sal B, and SB-431542 significantly decreased the secretion of these MMPs after being induced by TGF-β1. The values are as shown as folds *vs*. Control. **P < 0.01 *vs*. control; # #*P *< 0.01 *vs*. TGF-β1.

TGF-β1 can promote MMP-2 and MMP-9 activation in mesangial cells and fibroblasts, and augment tissue remodeling and fibrogenesis [26,27]. In this study, Zymographic analysis of conditioned media indicated that TGF-β1 induced pro-MMP-2 and its activation, as well as MMP-9 secretion. 1 μM and 10 μM of Sal B significantly decreased MMP-2/9 activities. (Figure 10).

### 7. Sal B treatment attenuated TGF-β1-induced TβR-I and Smad2/3 phosphorylation and up-regulated Smad7 expression in HK-2 cells

**Figure 11:**
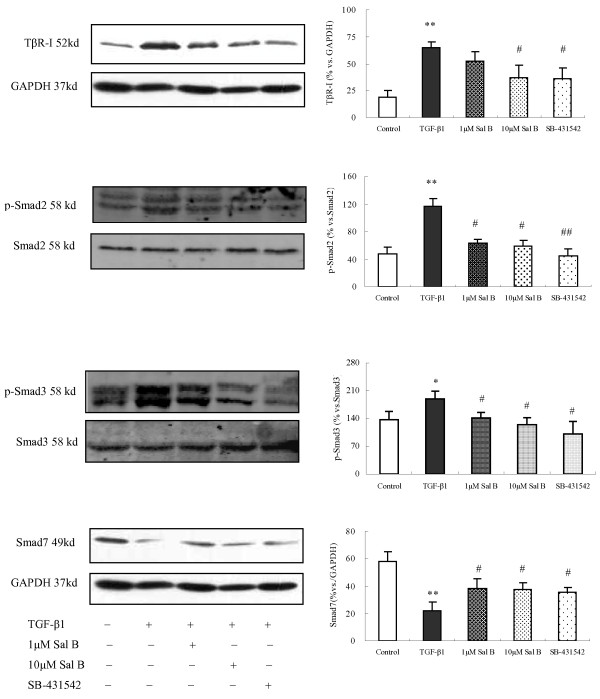
**Sal B attenuated TGF-β1-induced TβR-I and Smad2/3 phosphorylation and up-regulated Smad7 expression in HK-2 cells**. HK-2 cells were incubated with serum-free medium or treated with (1) 2.5 ng/ml TGF-β1, (2) 2.5 ng/ml TGF-β1 plus 1 μM of Sal B, (3) 2.5 ng/ml TGF-β1 plus 10 μM of Sal B, (4) 2.5 ng/ml TGF-β1 plus 10 μM of SB-431542 for 24 h. The expression of TβR-I, Smad2, Smad3, p-Smad2/3 and Smad7 was detected by Western Blot. There was no significant change between each group in Smad2/3, but the expression of phosphorylated Smad2/3 and TβR-I was significantly increased. Smad7 expression was decreased after incubation with TGF-β1. Treatment with 1 μM and 10 μM Sal B and SB-431542 significantly attenuated the TGF-β1-induced up-regulation of p-Smad2/3 and TβR-I and increased Smad7 expression. The values are represented as the density of p-Smad2/3, TβR-I or Smad7 *vs*. GAPDH (%). **P < 0.01 *vs*. control; #*P *< 0.05 *vs*. TGF-β1, # #*P *< 0.01 *vs*. TGF-β1.

As a mechanistic experiment for the effects of Sal B, we examined the influence of Sal B on the TGF-β1-Smads signaling, which is known to play a major role in TGF-β1-induced EMT. The results demonstrated that there was no significant change between each group in Smad2/3, but the expression of phosphorylated Smad2/3 and TβR-I was significantly increased after incubation with TGF-β1. Conversely, 1 μM and 10 μM of Sal B as well as SB-431542 treatments significantly attenuated the TGF-β1-induced up-regulation of p-Smad2/3 and TβR-I (Figure 11).

Likewise, the expression of Smad7, a negative regulator of TGF-β1 signaling, was reduced after TGF-β1 stimulation, while 1 μM and 10 μM of Sal B up-regulated its expression (Figure 11).

## Discussion

RIF is the common pathway in chronic renal diseases regardless of the initial cause. Therefore preventing RIF could slow down the pace of renal function deterioration [28]. Recent documents show that hepatocyte growth factor (HGF), angiotensin-converting enzyme inhibitors (ACEI), and erythropoietin all could antagonize RIF and improve renal function [6,29,30]. Traditional Chinese medicine has a rich history of treating chronic renal diseases over thousands of years, and some herbal medicine and extracts have proven to contain anti-renal fibrosis properties both *in vivo *[31] and *in vitro *[32]. It is well recognized that tubular epithelial cells could differentiate into myofibroblasts via a process referred to as EMT, which plays a pivotal role in renal interstitial fibrogenesis [21]. Since specific therapies of preventing the progression of chronic renal diseases are still lacking, seeking drug candidates or herbal compounds that can effectively prevent or inhibit EMT would represent one of the main strategies in the treatment of RIF.

Tubular EMT is a process during which renal tubular epithelial cells lose their epithelial phenotype and acquire new mesenchymal features and consists of four correlated steps with highly regulated mechanisms [21]: (1) Loss of epithelial cell adhesion through down-regulation of E-cadherin. (2) De novo α-SMA expression and actin reorganization, which may provide a structural foundation not only for defining the morphology of the transformed cells, but also for them to migrate, invade, and even acquire the capacity for contractility. (3) Disruption of tubular basement membrane, and (4) Enhanced cell migration and invasion. The latter 2 steps are mainly regulated by increased activities of MMP-2 and MMP-9 which would specifically break down native type IV collagen and laminin, the principal proteins found in the TBM, thereby disrupting TBM structural and functional integrity. Although recent evidence with genetic manipulation indicates that kidney epithelial cells do not become myofibroblasts *in vivo *[22], most documents still support the phenomenon of tubular EMT and its important role in renal fibrogenesis [21].

In this study, we found that α-SMA expression was increased significantly around tubules in the fibrotic kidney induced by HgCl_2_, using immmunohistochemistry staining, when compared with the normal kidney. In contrast, E-cadherin expression decreased around tubular areas. Although we have not confirmed if these α-SMA positive tubular cells come from the epithelium or whether they can synthesize collagen, the increased α-SMA expression around tubules and MMP-2 activity in kidney support EMT *in vivo*. Also, the *in vitro *results clearly show that human proximal tubular epithelial cells, represented by the HK-2 cell line, had undergone EMT after TGF-β1 incubation, as evidenced by the loss of E-cadherin, activation of α-SMA, acquisition of a spindle-like morphology, F-actin re-organization, and an increase in the activities of MMP2/9. More interestingly, Sal B could inhibit tubular EMT in fibrotic kidneys, as evidenced by the reversal of increased α-SMA and decreased E-cadherin expression, as well as preventing renal collagen deposition and fibrosis.

Vit E is a potential antioxidant agent and can prevent organ fibrosis [33-35]. In our previous study, we found that chronic mercury intoxication induced RIF with obvious characteristics of oxidative stress through the depletion of intracellular thiols [20]. Vit E exhibited anti-renal fibrosis effects through its mechanism of action, which protects the kidney from oxidative injury. Here we used Vit E as a control as well, and the results showed that Vit E also blocked EMT *in vivo*, but the effects were not as strong as Sal B. With the EMT cell model induced by TGF-β1 in HK-2, our data *in vitro *demonstrated that Sal B blocked EMT in HK-2 cells, and these results are consistent with our *in vivo *results as well as *in vitro *results from other reports [21]. However, in our experiment, reasonable dosages f Sal B (1 μM and 10 μM) were utilized, which produced no obvious toxicity, and were used effectively in other experiments [36]. In addition, SB-431542 [37], a potential and specific inhibitor of TβR-I kinase was used as an active control. The results revealed that Sal B, as well as SB-431542, significantly reversed EMT induced by TGF-β1 in HK-2 cells, as evidenced by the restoration of diminished E-Cadherin and CK-18 expression, blockage of α-SMA expression, cell morphology transformation, F-actin reorganization, and down-regulation of MMP-2 and MMP-9 activation. These data not only confirms Sal B's effects in preventing or reversing tubular EMT *in vivo *and *in vitro*, but also implies a mechanism of action involved with the regulation of MMP-2/9. MMP-2/9, in particular MMP-2, have pivotal roles in initiating renal fibrosis by degrading TBM components and disrupting its integrity [38]. It was reported that genetic expression of active MMP-2 in the renal proximal tubule is sufficient to generate the entire spectrum of EMT in the absence of superimposed injury. The rat RIF model induced by HgCl_2 _displayed a remarkable feature of oxidative stress, which could promote MMP-2 expression and activity [39]. Our previous studies showed that Sal B had anti-oxidant effects, and in this study, we found that Sal B can also inhibit MMP-2 activity in the diseased kidney and subsequently protect tubular basement membrane and prevent EMT and renal fibrosis.

TGF-β1 is the most potent inducer in initiating and completing the entire EMT course [4]. Its biological responses are primarily dependent on regulation via the TβR and Smads signaling pathway. TGF-β1 signals are transduced by transmembrane serine/threonine kinase type I and type II receptors, as well as intracellular mediators known as Smads [40-42]. Upon TGF-β1 stimulation, the activated TβR1 associates with R-Smads (Smad2 and Smad 3) and also phosphorylats them. Such phosphorylation of Smad-2/3 induces their association with their common partner Smad4 and subsequent translocation into the nucleus, where they control the transcription of TGF-β1 responsive genes [43,44]. As an inhibitory Smad, Smad 7 prevents or attenuates TGF-β signaling mainly through interaction with activated TβRI and prevention of R-Smad phosphorylation.

In this study, we found that the protein expression of TGF-β1 and TβRI and the phosphorylation levels of Smad 2 and Smad 3 were significantly increased in the fibrotic kidney, compared with ones in the normal kidney. In cultured HK-2 cells, TGF-β1 dramatically up-regulated TβRI expression and phosphorylated levels of Smad 2 and Smad 3, but down-regulated Smad7 expression, consistent with the data *in vivo*, and confirmed the characteristics of TGF-β/Smads signaling in EMT [44]. The down-regulation of Smad7 protein by TGF-β1 is most likely due to the promotion of Smad7 degradation [45]. In the rat model, Sal B and Vit E suppressed TGF-β1 and TβR-I expression and restored phosphorylation of Smad2 and Samd3 in fibrotic kidneys. Also, in cultured HK-2 cells, Sal B and SB-431542 significantly down-regulated the expression of TβR-I and p-Smad2/3, while restoring the diminished expression of Smad7 secondary to TGF-β1 stimulation. Although we did not find the action of Sal B to be dose dependent, the effects of 10 μM of Sal B are much more prominent than 1 μM of Sal B, and almost had a similar effect as the SB-431542 inhibitor, indicating that Sal B can antagonize TβR-I function as well as its protein expression. The above results indicate that Sal B can down-regulate TGF-β/Smads signaling pathway, via suppressing TGF-β1 expression, inhibiting TβR-I expression and function, down-regulating Smad 2/3 phosphorylation, and preventing the down- regulation of Smad7 by TGF-β1. As stated above, MMP-2 has a role in renal fibrosis, especially during early pre-fibrotic stage. However, MMP-2 and MMP-9 are not only stimulated by oxidative stress, but more importantly up-regulated by TGF-β1 through signal mediators such as Smads and ILK [21]. Our data also showed that MMP-2/9 activities increased in HK-2 cells after TGF-β1 incubation, but were reversed by Sal B and SB-431542. Therefore, the TGF-β1/Smads transduction pathway plays a key role in tubular EMT and Sal B's pharmacological mechanisms of action against EMT and renal fibrosis.

## Conclusions

Sal B can prevent tubular EMT in both the fibrotic kidney induced by HgCl_2 _as well as HK-2 cells triggered by TGF-β1. The mechanism of action of Sal B is closely related to the regulation of the TGF-β1 signaling pathway. It functions by inhibiting TGF-β1 expression, suppressing TβR-I expression and function, down-regulating Smad 2/3 phosphorylation, restoring the down- regulation of Smad7, and inhibiting the activity of MMP-2.

## Methods

### Reagents

HgCl_2 _was purchased from Tongren Chemical Reagent Factory, Guizhou, China. Vit E was purchased from Shanghai Yanan Wangxiang Pharmaceutical Co., Ltd, Shanghai, China. Recombinant human TGF-β1 (reconstituted with sterile 4 mM HCl containing 1 mg/mL BSA to a final concentration of 1 μg/mL) was obtained from R&D Systems, USA. SB-431542, a potent and specific inhibitor of TβR-I kinase was purchased from TOCRIS Bioscience, USA. AlarmarBlue™ was purchased from Nalgene Biosource International, Camarillo, CA, USA.

The Multiparameter Cytotoxicity 1 Kit was purchased from Thermo Fisher Scientific, Pittsburgh, PA, USA. Primary antibodies used in the study are listed in Table 1. Horseradish peroxidase-conjugated rabbit anti-mouse Ig and peroxidase-conjugated goat anti-rabbit Ig were obtained from Chemicon, Temecula, CA, USA. BCA Protein Assay Kit and SuperSignal West Pico Chemiluminescent Substrate were purchased from Pierce Chemical Company, USA. Cy3-labeled goat Anti-Mouse IgG (H+L) was provided by the Beyotime Institute of Biotechnology, Haimen, China. FITC-labeled goat Anti-Mouse IgG (H+L) was from Invitrogen Corporation, Carlsbad, CA, USA. RNeasy Mini Kit was purchased from Qiagen, Valencia, CA, USA. First strand cDNA synthesis kit was purchased from Fermentas, St. Leon-Roth, Germany. SYBR Green Real Time PCR Kit was from TakaRa Biotech, Tokyo, Japan. Primer oligonucleotide sequences specific for the real-time PCR are shown in Table 2, which were designed and synthesized by Sangon Biotech Inc, Shanghai, China. Gelatin was purchased from Amresco, Solon, OH, USA.

**Table 1:** Antibodies used in the study.

Antibody	Isotype	Suppliers	Cat. No	Dilution
α-SMA	Mouse IgG2a	Sigma	A2547	1:200
E-cadherin	Mouse IgG_2a_	BD Biosciences	610182	0.25 μg/ml
TGF-β1	mouse IgG1	R&D Systems	MAB240	5 μg/ml
TβR-I	Rabbit IgG	Cell Signaling Technology	#3712	1:1000
Smad2	Mouse IgG1	Cell Singaling Technology	#3103	1:800
Smad3	Rabbit IgG	Zymed Laboratories	51-1500	1:200
CK-18	Mouse IgG1	Chemicon International	MAB1600	1:200
p-Smad2	Rabbit IgG1	Cell Singaling Technology	#3101	1:200
p-Smad3	Rabbit IgG1	Cell Singaling Technology	#9520S	1:200
Smad3	Rabbit IgG	Epitomics	1735-1	1:2000
p-Smad3	Rabbit IgG	Epitomics	1880-1	1:1000
Smad2	Rabbit IgG	Epitomics	1736-1	1:1000
p-Smad2	Rabbit IgG	Abcam	Ab53100	1:300
Smad7	Rabbit IgG	Santa Cruz Biotechnology	SC-11392	1:100

**Table 2:** Primers used for real-time PCR.

Gene	Primer sequence (5'-3')	Gene bank number	Product length
E-cadherin	Forward: 5'-*CGC CGA GAG CTA CAC GTT CA*-3' Reverse: 5'-*TGT CGA CCG GTG CAA TCT TC*-3'	NM_004360	93 bp
β-actin	Forward: 5'-TGA CGA GGC CCA GAG CAA GA-3' Reverse: 5'-ATG GGC ACA GTG TGG GTG AC-3'	DQ237887	331 bp

### Drugs

Sal B was a generous gift from Prof. Da-yuan Zhu at the Shanghai Institute of Meteria Medica, Chinese Academy of Science, Shanghai, China. The purity of Sal B was 80% for the *in vivo *experiment and 98% for the *in vitro *experiment. Its molecular weight is 718, and its molecular formula is C_36_H_30_O_16_. The chemical structure of Sal B is shown in Figure 1. Sal B was dissolved in double distilled H_2_O at a concentration of 100 mM, filtered through a 0.22 μm filter, and stored at -70°C.

### *In Vivo *Experimental Design

Fourty-six Sprague-Dawley male rats, weighing 120 ± 10 g (SPF, Certificate No SCXK 2003-0003), were purchased from the Shanghai Laboratory Animal Center, Chinese Academy of Sciences. The rats were randomly divided into 4 groups: Normal (n = 8), Model (n = 12), Sal B Treatment (Sal B) (n = 14) and Vit E Treatment (Vit E) (n = 12). The renal interstitial fibrosis model was induced by the oral administration of HgCl_2 _at a dose of 8 mg/kg body weight once a day for 9 weeks [46]. Meanwhile, rats in the Sal B and Vit E groups were treated with Sal B at a dose of 10 mg/kg body weight and Vit E at a dose of 100 mg/kg body weight respectively once a day for 9 weeks. Rats were sacrificed 9 weeks after treatments and the kidneys removed. A portion of each kidney was fixed in 10% phosphate-buffered formalin for histological and immunohistochemical studies after paraffin embedding. The remainder was snap-frozen in liquid nitrogen and stored at -80°C for Hyp content determination and protein extraction. All experimental procedures were carried out in accordance with internationally accepted laboratory principles and all animals received humane care during the study with unlimited access to chow and water.

### Examination of Hyp Content in Renal Tissues

Hyp content of kidneys was assayed with HCl hydrolysis according to Jamall's methods [47]. Briefly, renal tissue samples weighing 100 mg were homogenized in 2.5 ml of ice-cold double-distilled water. After determining the total protein concentration in homogenates, 2 ml of homogenates were hydrolyzed with HCl (final concentration = 6 M) at 105°C for 18 h. Hydrolysates were filtered with 3 mm filter paper and dried at 40°C. The samples were then incubated with Ehrlich's solution (25% (w/v) p-dimethylaminobenzaldehyde and 27.3% (v/v) perchloric acid in isopropanol) at 50°C for 90 minutes and measured at the absorption of 558 nm (A_558_). All results were normalized by total protein concentration and calculated using a standard curve.

### Histologic Examination

The kidneys fixed in 10% phosphate-buffered formalin were embedded in paraffin and sectioned (3 μM thickness). The sections were then stained with Masson trichrome to assess for collagen fiber deposition.

### Immunohistochemical Examination

3 μm-thick sections were used for immunohistochemical examination. These sections were digested with pepsin at 37°C for 20 min, followed by incubation with 0.1% BSA in PBS for 30 min, and then incubated again with primary antibodies against E-cadherin (1:50) or α-SMA (1:100) at 37°C for 1 h. The sections were then incubated with FITC- or Cy3-labeled second antibodies at 37°C for 1 h, and images were obtained with a fluorescent microscope (Olympus).

### Cell Culture and Treatment

HK-2 cells were obtained from the Institute of Basic Medical Sciences Chinese Academy of Medical Sciences, and maintained in Dulbecco's modified Eagle's medium (DMEM) supplemented with 5% fetal bovine serum (FBS), 100 units/ml of penicillin, and 100 units/ml of streptomycin. The cells were cultured with 5% CO_2 _at 95% humidity. HK-2 cells were cultured in complete medium containing 5% FBS for 18 h. Thereafter, the cells were kept in serum-free medium or treated with (1) 2.5 ng/ml TGF-β1, (2) 2.5 ng/ml TGF-β1 plus 1 μM of Sal B, (3) 2.5 ng/ml TGF-β1 plus 10 μM of Sal B, or (4) 2.5 ng/ml TGF-β1 plus 10 μM of SB-431542 for 24 h.

### Cell Viability Assay

To determine cell viability, HK-2 cells were seeded at a density of 3,000 cells per well in 96-well cell culture plates. After growing for 18 h under normal growth conditions, the medium was replaced with DMEM containing different concentrations of Sal B and 10% alamarBlue (AB) solution. As a negative control, AB was added to the medium without cells. Medium only was used as blank control. The absorbance of test and control wells was read at 570 and 600 nm using a microplate reader (Molecular Devices) after 4 h, 12 h, and 24 h of incubation. The calculation of the percentage of AB reduction (%AB reduction) is as follows:

In the formula, A 570 and A 600 represent the absorbance of test wells at 570 and 600 nm, respectively. ^#^A570 and ^#^A600 represent the absorbance of negative control wells at 570 and 600 nm, respectively. *A570 and *A600 represent the absorbance of blank control wells at 570 and 600 nm, respectively.

### Assessment of cytotoxicity of Sal B with high content screening (HCS) assay

The cytotoxicity of Sal B on HK-2 cells was determined with the Multiparameter Cytotoxicity Kit 1. The principle of the assay is that live cells are labeled with a cocktail of fluorescent dyes that indicate the cellular properties of interest: (1) nucleus and its size, (2) cell membrane permeability status, (3) lysosome and other acidic organelles' pH and mass. All procedures were conducted according to the manufacturer's instruction. HK-2 cells were seeded at a density of 4 × 10^4 ^cells/ml in DMEM Medium plus 5% FBS in individual wells within a 96-well plate (approximately 4,000 cells/well). After culturing for 18 h, cells were incubated with 1 μM, 10 μM, or 100 μM of Sal B in serum-free DMEM for another 24 h. At 30 min before the completion of incubation, 1× MPCT1 Fluor Solution was added to each well. Then cells were fixed with pre-warmed Fixation Solution and washed twice with PBS. The plate was sealed and read with a HCS Kinetic Scan (Cellomics) immediately to acquire images.

### RNA Isolation, cDNA Synthesis, and Real-time RT-PCR

HK-2 cells were cultured in complete medium containing 5% FBS for 18 h. Thereafter, the cells were kept in serum-free medium or treated with (1) 2.5 ng/ml TGF-β1, (2) 2.5 ng/ml TGF-β1 plus 1 μM of Sal B, (3) 2.5 ng/ml TGF-β1 plus 10 μM of Sal B, or (4) 2.5 ng/ml TGF-β1 plus 10 μM of SB-431542 for 24 h.

Total cellular RNA was isolated using RNeasy Mini Kit according to the manufacturer's protocol. RNA concentration was determined spectrophotometrically, and its integrity was checked by agarose gel electrophoresis. cDNA was generated using 4 μg of total RNA in a final reaction volume of 20 μl by the RevertAid first-strand cDNA synthesis kit according to the manufacturer's protocol. Quantitative Real-time PCR was performed with a Rotor-gene 3000. Primers along with their sequences are listed in Table 2. PCR mixtures contained 1 μl of cDNA, 10 μl of SYBR^® ^Premix Ex Taq 2× and 0.25 μmol/L of forward and reverse primers, for a total volume of 20 μl. Reactions were started with a polymerase activation step at 95°C for 10 s followed by 40 cycles of 95°C for 5 s, 58°C for 15 s and 72°C for 10 s. Fluorescent data were acquired after each cycle. The absence of primer dimmers and non-specific products were verified after each run by melting curve analysis. The PCR mixture's relative quantity was calculated using a standard curve.

### Immunocytochemical Staining

Indirect immunofluorescence staining was performed. Briefly, HK-2 cells cultured on coverslips were washed with cold PBS twice and fixed with a cold mixture of methanol: acetone (1:1) for 10 min on ice. After extensive washing with PBS three times, the cells were permeated with 0.05% saponin for 15 min. The cells were then blocked with 5% bovine serum albumin in PBS buffer for 30 min at room temperature before incubation with primary antibodies against α-SMA (1:100) or CK-18 (1:100). To visualize the primary antibodies, cells were stained with FITC-conjugated secondary antibodies. After washing, cells were doublestained with Hoechst 33258 to visualize the nuclei. Stained cells were mounted with antifade mounting medium and viewed with a fluorescence microscope (Olympus).

### Western Blot Analysis

Kidney tissue and HK-2 cells were homogenized in lysis buffer (150 mM NaCl, 1% Nonidet P-40, 0.1% SDS, 50 mM Tris-HCl pH7.4, 1 mM EDTA, 1 mM PMSF, 1× Roche complete mini protease inhibitor cocktail, Roche PhosSTOP phosphatase inhibitor cocktail). The supernatants were collected after centrifugation at 10,000 g at 4°C for 15 min. Protein concentration was determined using a BCA protein assay kit. Equal amounts of protein were separated by 10% SDS gel electrophoresis (SDS-PAGE) under denaturing and non-reducing conditions and then transferred to a nitrocellulose membrane. The membrane was blocked with 5% nonfat milk in TBST at room temperature for 1 h and then incubated with primary antibody (Table 1) at 4°C overnight. After washing in TBST, the blots were incubated with horseradish-coupled secondary antibody. The signals were visualized using enhancence chemical luminescent (ECL) system.

### Gelatinase Activity Assay

MMPs activity in the kidney tissue and supernatant of cultured cells was assayed by gelatin zymography. Briefly, the kidney tissue was homogenized in lysis buffer (1 M Tris-HCl, 0.5 M NaCl, pH 7.0), the homogenate was centrifuged at 12000 × g for 30 min. For supernatant of cultured cells, conditioned media were collected and centrifuged at 13,000 × g for 5 min to remove any cell debris. The protein concentration was determined using a BCA protein assay kit. Aliquots of protein (15 μg protein/lane) were prepared by dilution into zymogram sample buffer (5×) consisting of 0.4 M Tris, pH 6.8, 5% SDS, 20% glycerol and 0.03% bromphenol blue. They were then separated by electrophoresis in 10% SDS-PAGE containing 1 mg/ml gelatin as a substrate in non-reducing conditions. After this process, the gel was rinsed with 50 mM Tris- HCl, 5 mM CaCl_2_, 1 μM ZnCl_2 _and 2.5% Triton-X 100 (pH 7.5) to remove SDS and then incubated at 30°C for 18 h with the same buffer minus Triton-X 100. After incubation, the gel was stained with 0.1% Coomassie Brilliant Blue G-250 in 30% methanol/20% acetic acid and destained with 30% methanol/10% acetic acid. Areas of digestion are visualized as non-staining regions of the gel, which represent gelatinase activity. The gel was scanned, and gelatinolytic activity was analyzed using Furi Gel Image software (Furi, Shanghai, China).

### Statistical Analysis

Values are expressed as mean ± SE. Statistical analyses were carried out using the one-way analysis of variance (ANOVA) followed by the least significant difference (LSD) post-hoc test. Values of *P *< 0.05 were considered significant.

## Abbreviations

α-SMA: alpha smooth muscle actin; AB: alamarBlue; ACEI: angiotensin-converting enzyme inhibitors; ANOVA: one-way analysis of variance; CK-18: Cytokeratin-18; DMEM: Dulbecco's modified Eagle's medium; EMT: epithelial-to mesenchymal transition; FBS: fetal bovine serum; HCS: high content screening; HGF: hepatocyte growth factor; HK-2 cells: Human proximal tubular epithelial cells; LSD: the least significant difference; MMPs: Matrix metalloproteinases; RIF: Renal interstitial fibrosis; ROS: reactive oxygen species; Sal B: Salvianolic Acid B; SM: Radix Salviae Miltiorrhizae; TBM: tubular basement membrane; TGF-β1: transforming growth factor-beta1; TβR-I: TGF-β type I receptor; Vit E: vitamin; ECL: enhancence chemical luminescent.

## Authors' contributions

C-HL conceived the study and established its initial design. Q-LW, J-LY, Y-YT and LS carried out the experimental work. Q-LW and Y-YT performed the statistical analysis. Q-LW and Y-YT prepared the manuscript, and C-HL made critical revisions. All authors read and approved of the final manuscript.
